# Functional and Structural Comparison of Pyrrolnitrin- and Iprodione-Induced Modifications in the Class III Histidine-Kinase Bos1 of *Botrytis cinerea*

**DOI:** 10.1371/journal.pone.0042520

**Published:** 2012-08-13

**Authors:** Sabine Fillinger, Sakhr Ajouz, Philippe C. Nicot, Pierre Leroux, Marc Bardin

**Affiliations:** 1 INRA UR1290, BIOGER CPP, Thiverval-Grignon, France; 2 INRA, UR407, Plant Pathology Unit, Montfavet, France; Nanjing Agricultural University, China

## Abstract

Dicarboximides and phenylpyrroles are commonly used fungicides against plant pathogenic ascomycetes. Although their effect on fungal osmosensing systems has been shown in many studies, their modes-of-action still remain unclear. Laboratory- or field-mutants of fungi resistant to either or both fungicide categories generally harbour point mutations in the sensor histidine kinase of the osmotic signal transduction cascade.

In the present study we compared the mechanisms of resistance to the dicarboximide iprodione and to pyrrolnitrin, a structural analogue of phenylpyrrole fungicides, in *Botrytis cinerea*. Pyrrolnitrin-induced mutants and iprodione-induced mutants of *B. cinerea* were produced *in vitro*. For the pyrrolnitrin-induced mutants, a high level of resistance to pyrrolnitrin was associated with a high level of resistance to iprodione. For the iprodione-induced mutants, the high level of resistance to iprodione generated variable levels of resistance to pyrrolnitrin and phenylpyrroles. All selected mutants showed hypersensitivity to high osmolarity and regardless of their resistance levels to phenylpyrroles, they showed strongly reduced fitness parameters (sporulation, mycelial growth, aggressiveness on plants) compared to the parental phenotypes. Most of the mutants presented modifications in the osmosensing class III histidine kinase affecting the HAMP domains. Site directed mutagenesis of the *bos1* gene was applied to validate eight of the identified mutations. Structure modelling of the HAMP domains revealed that the replacements of hydrophobic residues within the HAMP domains generally affected their helical structure, probably abolishing signal transduction. Comparing mutant phenotypes to the HAMP structures, our study suggests that mutations perturbing helical structures of HAMP2-4 abolish signal-transduction leading to loss-of-function phenotype. The mutation of residues E529, M427, and T581, without consequences on HAMP structure, highlighted their involvement in signal transduction. E529 and M427 seem to be principally involved in osmotic signal transduction.

## Introduction

Gray mould, caused by the fungus *Botrytis cinerea* Pers.:Fr (teleomorph *Botryotinia fuckeliana* (de Bary) Whetzel), is a severe disease affecting a wide range of economically important crops [1]. Chemical control is the main approach to limit the incidence of this pathogen. However, the efficiency of fungicides is under threat, because isolates of *B. cinerea* resistant to fungicides have been found to evolve rapidly [2], [3]. Biological control could be an alternative, or a complement, to chemical control because plant pathogens are considered to develop resistance to biocontrol agents less frequently than to fungicides [4]. Numerous biocontrol agents are effective against *B. cinerea* [5], [6]. For some of them, production of antibiotics is one of the putative mechanisms of action [7].

Pyrrolnitrin [3-chloro-4-(3-chloro-2-nitrophenyl) pyrrole], first isolated from *Burkholderia pyrrocina* [8], is an antibiotic with a broad-spectral antifungal activity [7]. It was also found in several other bacteria used as biocontrol agents against various fungal plant pathogens [9] including *B. cinerea* [10], [11], [12]. Under laboratory conditions, *B. cinerea* mutants resistant to pyrrolnitrin have recently been reported, suggesting a possible loss of efficacy of pyrrolnitrin-producing biocontrol agents [13]. Resistance to synthetic phenylpyrrole fungicides (e.g., fenpiclonil, fludioxonil), structural analogues of pyrrolnitrin, has also been reported in laboratory-induced mutants [14], [15], [16], [17]. The same studies revealed that mutants highly resistant to phenylpyrroles generally also displayed resistance to dicarboximide (e.g., iprodione) and aromatic hydrocarbon fungicides (e.g., dicloran). They also were found to be sensitive to osmotic stress [15], [17]. However, under field conditions such phenotypes have not yet been observed for *B. cinerea*. To date, no specific resistance to phenylpyrroles is known for field isolates of *B. cinerea*.

Molecular studies have shown that an osmosensing histidine kinase (HK) mediates resistance to dicarboximides and phenylpyrroles in *B. cinerea* [18], [19], [20], [21]. The same HK is also implicated in adaptation to adverse environmental conditions such as osmotic and oxidative stresses. Its essential role in the development and pathogenesis of *B. cinerea* [20], [22] may explain why strains highly resistant to dicarboximides and phenylpyrroles are not found under field conditions. In other fungal species, the role of homologous HKs in resistance to dicarboximides and phenylpyrroles has also been demonstrated [30], but in contrast to *B. cinerea*, specific resistance to fludioxonil was also found in field isolates [23], [24], [25], [26], [27], [28], [29], [30], giving rise to questions about the structure-function relationship of the Bos1 HK in *B. cinerea*.

Fungal osmosensing HKs belong principally to class III histidine kinases (according to the classification of Catlett and co-workers [31]) or, as in the case of *Saccharomyces cerevisiae*, which only has a unique HK,, to class VI HKs. Besides the typical HK domains the structural characteristics of the cytoplasmic class III HKs include five to six repeats of HAMP domains in their N-terminal half. HAMP domains are ubiquitous among eukaryotic and prokaryotic signal transduction (ST) proteins including histidine kinases, adenylate cyclases, methyl-accepting chemotaxis proteins and some phosphatases (reviewed in [32]). HAMP subunits contain two α helices, AS1 and AS2, bridged by a flexible connector of approximately 14 residues [33]. Each of the two helices is composed of a typical heptad repeat (a–g), with hydrophobic residues in positions a and d. Crystal structure and cysteine scanning studies of bacterial HAMPs revealed a typical four-helix bundle structure for HAMP dimers. Several putative mechanisms have been proposed for their role in signal transduction: piston movement, concerted rotation, or scissor-like movement between helices AS1 and AS2 or successive HAMPs [34], [35], [36], [37], [38]. In the case of eukaryotic proteins harbouring HAMP domains, in particular class III HKs, evidence of their role in ST has been brought to light by selecting osmo-sensitive and/or fungicide resistant mutants carrying point mutations in the HAMP domains [23], [24], [25], [26], [27], [28], [29], [30]. More recently, *in vitro* mutagenesis of successive HAMP domains of the *Debaromyces hansenii* DhNik1 protein has been used [39]. Meena and colleagues [39] proposed the first functional model of a class III HK based on osmosensing in the heterologous system *S. cerevisiae*. According to their model of this five-HAMP-containing HK, HAMP4 cross-links to HAMP5 under high osmolarity, thereby inhibiting histidine kinase activity. Under normal osmolarity, HAMP4 is kept away from HAMP5 through its binding to HAMP1-3, thereby maintaining histidine kinase activity. In the case of the *B. cinerea* class III HK Bos1, six HAMP domains have been previously identified [19], leading us to think that the signal transduction mechanism could be different.

Dicarboximide and phenylpyrrole fungicidal signals are transduced through the Bos1 HK in addition to osmosensing [19], [22]. In this study, from a functional point of view, we set out to investigate if these different fungicides induce mutations conferring different resistance phenotypes. Moreover, we asked what impact the resistance to fungicides has on fitness cost. From a molecular point of view, we examined the impact of the mutations on HAMP structure and its correlation with differential signal transduction.

## Materials and Methods

### Fungal isolates, culture conditions and mutant selection

Five single-spore isolates of *B. cinerea* (namely BC1, BC21, BC25, BC26 and H6) were selected from a collection maintained in the laboratory. BC1, BC25, BC26 and H6 were isolated from tomato between 1989 and 1991 and BC21 was isolated on strawberry in 1991. The choice was made on the basis of differences in patterns of resistance to 14 fungicides representing 11 chemical groups [12] and of differences in aggressiveness to tomato plants and apple fruits [13]. All isolates were sensitive to pyrrolnitrin. BC1 and BC26 were resistant (LR) to dicarboximides.

For DNA isolation, 10^7^ spores were harvested and used to inoculate 100 ml liquid yeast-sugar-salt medium (YSS, 2 g L^−1^ of yeast extract, 10 g L^−1^ of glucose, 2 g L^−1^ of KH_2_PO_4_, 1.5 g L^−1^ of K_2_HPO_4_, 1 g L^−1^ of (NH_4_)_2_SO_4_, 0.5 g L^−1^ of MgSO_4_ 7H_2_O) and grown for 16 hours at 23°C with 150 rpm shaking.

Twenty successive conidial transfers were performed with the 5 isolates of *B. cinerea* on increasing doses of pyrrolnitrin and iprodione as described previously [13], [40]. For each transfer, plates were incubated for 14 days at 21°C. As a control, twenty successive conidial transfers were performed on unsupplemented PDA medium. For each isolate, the whole experiment was carried out three times independently, aiming to provide three lineages of 20 transfers produced under selection pressure and three independent control lineages produced on PDA. To facilitate reading and avoid lengthy repetitions in the rest of this paper, control isolates produced on PDA medium and isolates produced in presence of pyrrolnitrin or iprodione will be labelled GnC, GnP and GnI respectively, where n indicates the transfer rank in the lineage. All isolates and mutants were maintained in stock cultures at −20°C in a 0.06 M phosphate buffer containing 20% (V/V) glycerol until they were used for phenotypic and genotypic characterizations. The biological and sequencing data are not available for mutants BC1G20P3, BC25G20P2, BC25G20P3, BC26G20P3 and H6G20I1 as they were lost before experiments could be achieved.

### Antifungal assays

To determine the sensitivity to the antibiotic pyrrolnitrin, the mycelial growth was measured on PDA medium containing different concentrations of pyrrolnitrin as described previously [13]. We assessed the effect of iprodione, fenpiclonil and fludioxonil (technical grade quality, kindly provided respectively by BASF Agro, Germany and Syngenta Crop Protection AG, Switzerland) on spore germination and germ tube elongation as previously described [15]. The experiments were all repeated three times independently per lineage, each with three replicate plates. For each combination of strain/antifungal molecule, the concentration leading to 50% decrease in mycelial growth or germ-tube elongation (EC_50_) was estimated by linear regression analysis of fungal development (as percentage of control values) [15]. To simplify the reading of the manuscript, we adopted the following nomenclature based on the established EC_50_ values. The ratio between the EC_50_ value of a tested strain and the mean EC_50_ value of fungicide-sensitive strains (BC21, BC25, and H6) were calculated for pyrrolnitrin, fludioxonil, fenpiclonil, and iprodione. For all compounds but iprodione, the strains were then considered as sensitive (S) if the ratio was between 0.5 and 2, slightly resistant (LR) between 2 and 20, moderately resistant (MR) between 20 and 100 and highly resistant (HR) for ratios over 100. In the case of iprodione, we adopted the classification LR (ratio between 2 and 10), MR (ratio between 10 and 20) and HR (ratio>20) from previous publications [15], [18], [19]. Resistance profiles to 10 other fungicides belonging to 9 different chemical families were established at discriminatory concentrations as already published [12].

Based on previous work, the resistance profiles of B05.10 transformants were established at the mycelial growth stage on discriminatory fungicide concentrations as follows. A transformant was considered as Iprodione LR if it was able to grow at 2.5 µg ml^−1^ but not at 25 µg ml^−1^, and it was considered Iprodione HR if it grew at 25 µg ml^−1^. It was considered as Phenylpyrrol LR if it grew on fludioxonil or fenpiclonil at 0.1 µg ml^−1^ but not at concentrations >1 µg ml^−1^ and Phenylpyrrol HR if it grew on fludioxonil or fenpiclonil at 5 µg ml^−1^. The discriminatory concentrations used for pyrrolnitrine LR and HR phenotypes were 0.05 µg ml^−1^ and 0.5 µg ml^−1^ respectively.

### Osmotic stress assay

To determine *in vitro* sensitivity to osmotic stress, PDA or YSS plates were supplemented with 0.5 M or 1 M of either NaCl or sorbitol. The plates were then inoculated with 5-mm mycelial plugs taken from the periphery of a 4-day-old colony of the various tested strains. Cultures were incubated for 4 days at 21°C and growth was compared relative to that on PDA or YSS control plates as no growth, reduced or comparable growth. Strains showing comparable growth on 0.5 M osmolytes were considered as resistant. Those with reduced growth on 0.5 M and no growth on 1 M osmolytes were classified as osmosensitive. Two to three replicate plates were realized for each treatment.

### *In vitro* and *in planta* estimation of fitness cost of the iprodione-induced mutants

Different fitness parameters were used to compare the strains. *In vitro* estimation of fitness was based on mycelial growth and spore production on PDA medium as described previously [13]. Statistical analyses were performed separately for each strain and each type of fitness parameter. In these analyses, we used the ANOVA module of Statistica software to test both a possible lineage effect and a number-of-transfer effect. When a significant effect was observed, the multiple comparison test of Student-Newman-Keuls was used to compare the means. To estimate fitness *in planta*, the aggressiveness of the various isolates was assessed on apple fruits cv. Golden Delicious (for all isolates) and on tomato cv. Monalbo plants (for strains BC1 and BC21), as previously described [13]. In addition, spore production was assessed on tomato plants for these two strains as follows. After 7 days of incubation in a growth chamber (21°C, relative humidity >90%, photoperiod of 14 hours), the stem segments carrying lesions were excised and placed in 5-mL water aliquots to collect spores. The concentration of the spore suspension was evaluated using a haematocytometer. Sporulation was then computed as the numbers of spores produced per mm of stem lesion. The experiments were all repeated two to three times independently per lineage, each with three replicate plants or fruits. To take into account the kinetics of disease development for each isolate, we computed the area under the disease progress curve (AUDPC) as already described [41]. Statistical analyses were performed separately for each isolate on the AUDPC values.

### DNA manipulations

Genomic DNA was extracted from approximately 1 g of fresh fungal mycelium using a Sarcosyl-based protocol [42]. As most iprodione resistant mutants from *B. cinerea* resulted in modification of the class III histidine kinase Bos1 involved in osmosensing [19], [22] the corresponding *bos1* gene from *B. cinerea* was sequenced. The primer pair bos1-F3 and bos1-R2 (Table S2), amplifying the fragment encoding the N-terminal half of the Bos1 histidine kinase between residues 192 and 741 harboring the HAMP domains, or the primer pair His1 and bos1-R5 (Table S2) amplifying the C-terminal half of the Bos1 protein (residues 593 to the end), were used. All primers used for PCR amplifications and DNA sequencing are listed in Table S2. The 3′ half of the gene (corresponding to the C-terminal half of the protein) was only sequenced in the absence of mutations in the 5′ half. PCR reactions were carried out with high-fidelity DNA polymerase (Phusion, Finnzymes) and were gel-purified prior to the sequencing reactions. The resulting sequences were quality analysed (PHRED>20) and aligned to the reference sequences using the CodonCode Aligner software (CodonCode Corp., Dedham, MA). The *bos1* sequences of all isolates are available from Genbank under the accession numbers JX192607–JX192631.

### *bos1* site directed mutagenesis by homologous recombination in the B05.10 wild-type strain

2.5 kb fragments of mutated *bos1* alleles to be studied were amplified on genomic DNA of the sequenced strains listed in Table 1 using the primers bos1_promLP2 and bos1-R2 listed in Table S2 using proofreading Taq polymerase (Phusion, Finnzymes). The 5′ extremities of the primers are not located in the *bos1*-coding region, but in the promoter- and intron sequence respectively, in order to minimize the impact of mutations introduced during the recombination at the fragment's extremities. The PCR amplicons were gel-verified prior to purification with the Nucleo spin extraction kit (Macherey & Nagel, Düren, Germany). Protoplasts of the *B. cinerea* reference strain B05.10 [43] were prepared and transformed as described by Levis et al. [44] with 5–7 µg of each purified PCR product. Protoplasts were spread on non-selective, isotonic medium and incubated for 24 h at 23°C. The plates were then overlaid with YSS medium containing 3 µg ml^−1^ iprodione as selective agent and incubated at 23°C for an additional 48 h.

**Table 1:** Phenotypes and changes in Bos1 peptide sequence in pyrrolnitrine- and iprodione-induced mutants.

Isolate	Transfer generation^a^	Phenotype^b^				Bos1 peptide sequence	HAMP n°
		Osmotic stress	Pyrrolnitrin	Phenylpyrroles	Iprodione		
BC1	G0	R	S	S	LR	I365S	3
	G20C	R	S	S	LR	nd	
	G20P1	S	HR	HR	HR	I365S, G311R	3,2
	G20P2	S	HR	HR	HR	I365S, G311E	3,2
	G20I1	S	MR	MR	HR	I365S, E692K	3,6
	G20I2	S	HR	MR	HR	I365S, E692K	3,6
	G20I3	S	LR	MR	HR	I365S, V239F	1
BC21	G0	R	S	S	S	wt	
	G20C	R	S	S	S	nd	
	G20I1	S	HR	HR	HR	G278D	2
	G20I2	S	HR	LR	HR	G323C	2
	G20I3	S	nd	nd	HR	G323C	2
BC25	G0	R	S	S	S	wt	
	G20C	R	S	S	S	nd	
	G20P1	S	HR	HR	HR	G415D	3
	G20I1	S	HR	MR/HR	HR	wt	
	G20I2	S	MR	MR	HR	A493T	4
	G20I3	S	MR	HR	HR	A493T	4
BC26	G0	R	S	S	LR	I365S	1
	G20C	R	S	S	LR	nd	
	G20P1	S	HR	HR	HR	I365S, T581P	3,5
	G20P2	S	HR	HR	HR	I365S, T581P	3,5
	G20I1	S	LR	MR	HR	I365S, E529A	3,4
	G20I2	S	HR	nd	HR	I365S	3
	G20I3	S	MR	MR	HR	I365S, E529A	3,4
H6	G0	R	S	S	S	wt	
	G20C	R	S	S	S	nd	
	G20P1	S	HR	HR	HR	G81STOP, A157T	nonsense
	G20P2	S	HR	HR	HR	G81STOP, A157T	nonsense
	G20P3	S	HR	HR	HR	G81STOP, A157T	nonsense
	G20I2	S	MR	LR	HR	I365S, M427T	3
	G20I3	S	MR	LR	HR	I365S, M427T	3

a G0 is the wild-type parent isolate, G20C is the 20^th^ transfer generation produced on PDA medium (control), G20P is the the 20^th^ transfer generation produced on PDA supplemented with pyrrolnitrin and G20I is the 20^th^ transfer generation produced on PDA supplemented with iprodione.

b S: sensitive, LR: low resistance, MR: moderate resistance, HR: high resistance, according to the resistance level classification explained in the Materials & Methods section and EC_50_ values indicated in Table S1.

nd: not determined.

Transformant colonies were isolated twice on selective YSS medium containing either 3 µg ml^−1^ iprodione or 0.3 µg ml^−1^ fludioxonil prior to DNA extraction. The presence of the introduced mutation and the purity of the transformants were verified by sequencing the *bos1* fragment amplified with primers bos1-F1 and bos1-R1 (Table S2, located outside the fragment used for transformation). Heterokaryons were further purified on selective medium until purity of the introduced mutant allele was achieved.

### HAMP structure modeling

**Figure 1:**
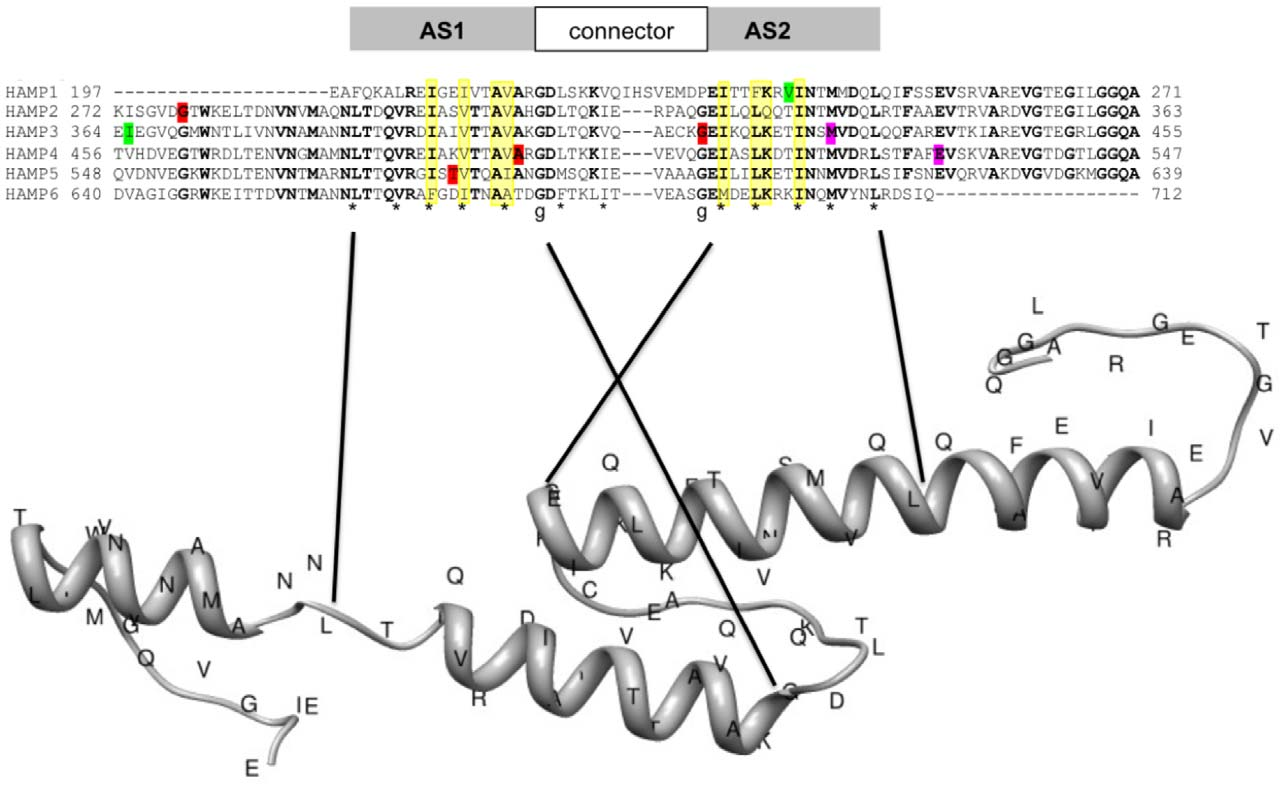
Sequence conservation and model structure of Bos1 HAMP domains. The amino-acid sequences of the six HAMP domains of the Bos1 protein were aligned with Clustal W. Amino acids identical over 80% are in bold. In the bottom panel “*” denotes hydrophobic core residues at critical heptad positions and hydrophobic residues of the connector. “g” corresponds to the conserved glycine residues of the connector motif (according to [35] and [32]). Interacting residues derived from *in silico* structures are highlighted in yellow. Mutated residues are shaded with the following color code according to the phenotypes indicated in Table 3: red = HR to iprodione and phenylpyrroles, osmosensitivity; green = LR to iprodione; purple = MR to iprodione and osmosensitivity. The structure of the HAMP domain 3 was predicted on the SWISS-MODEL server using the alignment mode (for details see text). Portions of the HAMP sequences involved in the typical HAMP structures AS1, AS2 and the connector are indicated above the sequence.

All homology modeling analyses were performed on the SWISS-MODEL workspace [45]. Prior to modeling, we specified the positions of the HAMP domains of the Bos1 protein with an InterPro Domain Scan and HMM scan [46] on the protein sequence (BAB69486). The six identified HAMP domains including their connecting sequences were aligned using the ClustalW algorithm [47]. The coordinates from these newly defined HAMP domains (Figure 1) differ from those previously published [19]. In order to identify the best structural model template, a non-iterative blast search with the Bos1 peptide sequence covering residues 190 to 720 was performed against the SWISS-MODEL template library. For the fraction comprised between position 195 and 524, template scores >100 were found with the structure 3lnrA established on the aerotaxis receptor Aer2 of *Pseudomonas aeroginosa* [35] and for the fraction comprised between 548 and 678 with the structure 1qu7A, established on the chemotaxis receptor TarH of *Escherichia coli* [48], with p-values<10^−19^. For reasons of homogeneity of our analysis, we also used the alignment of the C-terminal section (572–708) to the structure 3lnrA (score = 45; p-value = 2 10^−9^).

HAMP domain sequences (wild-type or mutant peptide sequences) with the coordinates presented in Figure 1 were aligned to the 3lnrA peptide sequence (GI:295789465) using MUSCLE [49]. The alignments were submitted to the SWISS-MODEL server using the alignment modes. Rather than simply replacing the candidate amino acid in a model of wild-type HAMP, each sequence was modeled separately. All models were visualized using the molecular graphics program Chimera [50].

## Results

### *In vitro* sensitivity profiles of pyrrolnitrin- and iprodione-induced mutants to pyrrolnitrin, fungicides and osmotic stress

The five *B. cinerea* parental strains used in this study were all susceptible to pyrrolnitrin and to the phenylpyrrole fungicides fludioxonil and fenpiclonil. Towards the dicarboximide iprodione they showed different behaviours, either sensitive (EC_50_ between 1 and 2.5 µg ml^−1^ in the case of BC21, BC25, H6) or displaying LR resistance (EC_50_ between 6 an 10 µg ml^−1^ for BC1 and BC26, Table S1). This last category corresponds to the previously described ImiR1 phenotype [15]. We then selected three iprodione-resistant mutant lines per parental strain by twenty successive conidial transfers on medium containing high concentrations of iprodione (steadily increasing from 5 to 200 mg ml^−1^). After conidial isolation, the three mutants per parental strain – named G20I1 to G20I3 – were tested for their susceptibility to pyrrolnitrin, to iprodione, and to the phenylpyrroles fenpiclonil and fludioxonil in parallel with the analysis of pyrrolnitrin-induced mutants – named G20P1 to G20P3 – selected from the same parental strains (described in [13]). The results summarized in Table 1 (detailed in Table S1) show that the pyrrolnitrin-induced mutants (G20P) exhibited high resistance levels to pyrrolnitrin (EC_50_>4.5 µg ml^−1^), but also to phenypyrroles (EC_50_>8 µg ml^−1^) and to iprodione (EC_50_>25 µg ml^−1^), whereas the iprodione-induced mutants (G20I), highly resistant to iprodione (EC_50_>25 µg ml^−1^), displayed variable resistance levels to pyrrolnitrin, in most cases much lower than the corresponding G20P mutants (EC_50_ reaching from 0.03 to over 0.5 µg ml^−1^). Similarly, the resistance levels to the phenylpyrroles fenpiclonil and fludioxonil were extremely variable for the iprodione induced mutants (EC_50_ between 0.1 and over 10 µg ml^−1^). In a few cases, resistance levels to pyrrolnitrin did not correlate with those observed on phenylpyrrole fungicides (Table 1, Table S1). For example, the mutant BC21G20I2, highly resistant to pyrrolnitrin, displayed only a low resistance level to the phenylpyrroles fludioxonil and fenpiclonil. The opposite was observed for BC1G20I3 with moderate resistance to phenylpyrroles and low resistance to pyrrolnitrin. The detailed pattern of resistance of the 5 parental strains (G0) to other fungicides is already known [12]. The selected mutants (G20C, G20P, G20I) displayed the same profiles as the parental strains on all these fungicides (data not shown).

Finally, all G20P and G20I mutants were highly sensitive to osmotic pressure resulting from 1 M sodium chloride and 1 M sorbitol compared to the wild-type parents G0 (data not shown).

### Fitness of iprodione-induced mutants

We have previously observed that high level of resistance to pyrrolnitrin is correlated to a high fitness cost [13], [51]. Since the iprodione-induced mutants displayed low levels of resistance to pyrrolnitrin, we investigated if they could also be associated with a fitness penalty.

Different parameters of fitness were studied for the parental strains G0, the control strains G20C and for all the lineages of the iprodione-induced mutants G20I. These fitness parameters included mycelial growth on PDA medium, spore production (assessed on PDA medium and on tomato plants for isolates derived from BC1 and BC21) and aggressiveness on apple fruits and on tomato plants (for isolates derived from BC1 and BC21). The average mycelial growth rate of the iprodione-induced mutants G20I was significantly reduced for each strain compared to G0 and G20C (56% reduction, *P*<0.0001 for strain BC1, 29% reduction, *P* = 0.0005 for BC21, 42% reduction, *P*<0.00001 for BC25, 38% reduction, *P*<0.0001 for BC26 and 10% reduction, *P* = 0.0001 for H6) (Table 2). Spore production of the iprodione-induced mutants G20I was significantly reduced on PDA medium (between 75 and 96%) for all lineages compared to G0 and G20C (*P* = 0.002, 0.047, 0.027, 0.0007 and 0.002 for BC1, BC21, BC25, BC26 and H6, respectively) (Table 2). It was also greatly reduced on tomato plants (between 80 and 87%) for all mutant lines of BC1 and BC21 strains.

**Table 2:** Comparison of fitness parameters between the iprodione-induced mutants and the parental strains.

		Mycelium	Sporulation	Sporulation on tomato stem	Aggressiveness (AUDPC)	
Strain	Transfer generation^a^	daily radial growth (mm/day)^b^	×10^6^ spores/Petri plate^c^	×10^3^ spores/mm lesion^d^	Apple fruit	tomato plant
BC1	G0	39.0 a^e^	144 a	234 a	178 a	125 a
	G20C	37.2 a	157 a	183 a	138 a	97 a
	G20I1	23.7 c	35 b	48 b	12 b	9 b
	G20I2	25.3 c	22 b	41 b	15 b	7 b
	G20I3	27.7 b	17 b	42 b	31 b	10 b
BC21	G0	35.3 a	57 a	207 a	165 a	66 a
	G20C	33.8 a	51 a	163 a	135 a	51 a
	G20I1	20.4 b	4 b	36 b	14 b	8 b
	G20I2	20.9 b	2 b	27 b	34 b	12 b
	G20I3	21.7 b	5 b	30 b	10 b	9 b
BC25	G0	28.0 a	94 a		120 a	
	G20C	27.5 a	98 a		102 a	
	G20I1	18.8 b	14 b		16 b	
	G20I2	15.9 c	3 b		24 b	
	G20I3	14.6 c	6 b		25 b	
BC26	G0	22.0 a	77 a		102 a	
	G20C	22.3 a	66 a		111 a	
	G20I1	15.3 c	24 b		11 b	
	G20I2	20.0 b	14 b		16 b	
	G20I3	15.0 c	16 b		6 b	
H6	G0	28.5 a	97 a		76 a	
	G20C	27.7 a	91 a		82 a	
	G20I2	18.9 b	6 b		8 b	
	G20I3	18.4 b	3 b		8 b	

a G0 is the wild-type parent isolate, G20C is the 20^th^ transfer generation produced on PDA medium without iprodione (control) and G20I is the 20^th^ transfer generation produced on PDA supplemented with iprodione.

b Daily radial growth rate between the 3^rd^ and 4^th^ day after inoculation.

c Spore produced on PDA medium 14 days after inoculation.

d Spore produced on tomato stem 7 days after inoculation.

e Data are means of two to three independent repetitions. For each isolate, means within a column followed by the same letter were not significantly different (ANOVA, α = 0.05; Newman–Keuls test).

For each of the five strains tested, the iprodione-induced mutants G20I were significantly less aggressive on apple fruits than the parental strains G0 and the control G20C (Table 2). On tomato plants, a decrease in aggressiveness was also observed with the iprodione-induced mutants G20I of strains BC1 and BC21 compared to G0 and G20C. Taken together, these results showed for all strains, a reduced *in vitro* growth rate, a severely reduced sporulation rate, and 5 to 10 fold reduction in lesion development. No significant lineage effect for the fitness parameter of any of the iprodione-induced isolate was observed (*P*>0.05) and no correlation could be drawn between the levels of resistance to pyrrolnitrin and the degree of fitness modification.

### Sequence analysis of the *bos1* gene in pyrrolnitrin- and iprodione-induced mutants

As underlined above, mutants selected on pyrrolnitrin or iprodione showed both comparable phenotypes on the basis of fungicide cross-resistance profiles, and clearly identical phenotypes in terms of sensitivity to hyperosmotic conditions. This suggests similar molecular mechanisms for resistance. Resistance to the dicarboximide iprodione and/or to the phenylpyrrole fludioxonil is conferred in many fungi by mutations of the osmosensing histidine kinase, which is orthologous to Bos1 in *B. cinerea* (reviewed in [52]). We therefore sequenced the *bos1* gene in the G20P and G20I induced mutants and compared it to that of the parental G0 strains. We focused on the HAMP domains involved in signal transduction (reviewed in [32]). The amino acid changes observed are listed in Table 1.

The parental strains BC1G0 and BC26G0 that are resistant to low iprodione concentrations (ImiR1) harbor a modification of isoleucine at position 365 to serine (I365S). The same mutation was observed in all mutants derived from BC1 and BC26, but also in the iprodione resistant mutants derived from strain H6 (H6G20I2, H6G20I3). Other mutations in this N-terminal half of the Bos1 protein were selected in pyrrolnitrin- and iprodione-induced mutants displaying high resistance levels to iprodione (HR, Table 1). These mutations were more or less equally distributed over the six HAMP domains (Table 1). Only for two resistant mutants (BC25G20I1 and BC26G20I2) we did not detect any change in the Bos1 peptide sequence compared to the parental strain. All pyrrolnitrin-induced mutants derived from the H6 strain carry a nonsense mutation in the *bos1* gene at codon 81 leading to precocious stop of translation. It is interesting to note that in some cases, independently selected mutants from the same isolate displayed the same mutation (Table 1). However, identical mutations were selected only by identical treatments (pyrrolnitrin or iprodione, respectively).

### *bos1* site-directed mutagenesis

Since the above analysed mutants had been selected in 20 conidial transfers on steadily increasing iprodione or pyrrolnitrine concentrations, we could not exclude the inadvertent selection of mutations – even outside *bos1* – in addition to those identified by sequencing. We therefore chose to introduce the following 11 modifications into a *bos1* wild-type strain, the sequenced reference strain B05.10 [43]: I365S, I365S G331R, I365S E692K, I365S V239F, I365S T581P, I365S E529A, I365S M427T, G278D, G323C, G415D, A493T. We transformed the B05.10 wild-type strain with 2.5 kb fragments covering the 5′ half of each *bos1* allele obtained by PCR on the corresponding mutant strains (for details see Materials and Methods). After 24 h protoplast regeneration, transformants were selected on iprodione at 3 µg ml^−1^. The absence of spontaneous mutations was validated by the negative control, without exogenous DNA. For each PCR fragment used we obtained from 30 to over 100 iprodione-resistant transformants. Fifteen transformants per allele were picked and isolated twice on selective medium. The insertion of the desired mutation at the native *bos1* locus was verified by sequencing after PCR amplification with the primers bos1-F1 and bos1-R1 (Table S2) located up- and downstream respectively of the PCR fragment used for transformation. Only homokaryotic transformants with validated *bos1* sequence - unmodified except for the studied mutation – were used for further analyses. We observed a high mutation rate within the transformed *bos1* fragment. We therefore could retain only few transformants; three mutations could not be validated using this approach (i.e., G323C, G311R, E692K).

The transformants with validated *bos1* sequence (n = 1 or 2 as indicated in Table 3) were tested for sensitivity to iprodione, to phenylpyrrole fungicides and to pyrrolnitrine. We also established their sensitivity to hyperosmolarity on NaCl and sorbitol. The observed phenotypes are listed in Table 3. Compared to the complex phenotypes observed for the G20P and G20I isolates described above and in Table 1, we can classify the *bos1* mutant phenotypes into three categories: 1/ those displaying high or moderate resistance levels to all tested fungicide categories associated with sensitivity to hyperosmolarity (i.e. G278D, G415D, I365S T581P, A493T); 2/ those displaying weak resistance to the dicarboximide iprodione, susceptibility to the phenylpyrroles including pyrrolnitrine and no osmosensitivity (i.e., I365S, I365S V239F), and 3/ mutants resistant to the dicarboximide, but not to phenylpyrroles and sensitive to hyperosmolarity (i.e. I365S E529A, I365S M427T). Comparing mutant phenotypes between Tables 1 and 3, the following mutations showed the expected phenotypes: I365S, G278D, G415D, A493T, I365S T581P. The *bos1* mutations I365S V239F, I365S E529A, I365S M427T introduced through site-directed mutagenesis lead to phenotypes diverging from those of the iprodione-induced mutants they have been identified in.

**Table 3:** Modifications in Bos1 peptide sequence and associated phenotypic and structural changes.

Bos1 peptide sequence	Phenotype^a^				HAMP^b^	AS^c^	aa change^d^	structural changes^e^	model (HAMP n°)
	Osmotic stress	Pyrrolnitrin	Phenylpyrroles	Iprodione					
I365S (n = 2)	R	S	S	LR	3	out	NH to PU	Yes	HAMP3: start of AS1 and AS2 helical structures displaced . Interacting residues different from wt HAMP3.
I365S, V239F	R	S	S	LR	3, 1	2	ali to aro	Yes	HAMP1: middle helix of AS2 dist. to V239 lost, but most interactions maintained.
G278D	S	HR	HR	HR	2	1	NH to -	Yes	HAMP2: helix prox. to G311 lost, new helical region in AS1 incl. D278
G415D	S	HR	HR	HR	3	2	NH to -	Yes	HAMP3: severe impact on AS2 helixes
A493T	S	MR	MR/HR	HR	4	1	NH to PU	Yes	HAMP4: destruction of AS1's table-3-captionterminal helix
I365S, T581P	S	HR	HR	HR	3, 5	1	PU to NH	No	HAMP5: no visible changes
I365S, E529A (n = 2)	S	S/LR	S(Flu)/LR(Fen)^f^	MR	3, 4	2	PU to NH	No	HAMP4: no visible changes
I365S, M427T (n = 2)	S	S	S/LR	MR	3	2	NH to PU	Yes	HAMP3: similar to HAMP3^I365S^

a S: sensitive, LR: low resistance, MR: moderate resistance, HR: high resistance, according to the resistance level classification explained in Materials & Methods. nd: not determined.

b modified HAMP; numbering according to the peptide modifications cited in the first column.

c AS: amphiphilic helix in HAMP.

d Characteristics of amino acids: PU = polar uncharged, NH = non polar hydrophobic, + = positively charged, − = negatively charged, ali = aliphatic, aro = aromatic; na: not applicable.

e Structural changes as presented in Figure 2 and explained in the text.

f Flu = Fludioxonil; Fen = Fenpiclonil.

### HAMP structure modelling

In order to get a clearer picture of the consequences on HAMP structures induced by the amino acid replacements, we conducted homology structure modelling on the Bos1 wild-type and mutant HAMP domains. Prior to performing the model analysis, we defined the HAMP domains in the Bos1 protein relative to the currently available domain databases rather than those published in 2002 [19] in order to adjust the coordinates of Bos1 HAMPs to those with established crystal structures [38], [51]. An HMM scan [53] revealed six HAMP domains with HMM scores >10 and c-values>0.01 (data not shown). We based our HAMP domain alignment presented in Figure 1 on the coordinates obtained in this search. The central core of the six repeats shows the typical feature of HAMP domains (reviewed in [32] and [54]): two α helices, AS1 and AS2 with heptad pattern of hydrophobic residues, as highlighted by the asterisks in Figure 1; the connector between AS1 and AS2 shows the conserved glycine residues (g) at each extremity in addition to the two hydrophobic residues indicated by “*” in Figure 1. This last feature is in agreement with the classification of divergent HAMPs proposed by Dunin-Horkawicz & Lupas [54]. The absence of the Pro6 residue conserved in canonical HAMPs also fits the classification as divergent HAMP.

Considering the different mutations selected and validated by mutagenesis, all HAMP domains were affected either in AS1 or AS2. The mutations related to high levels of cross-resistance to dicarboximides and phenylpyrroles affect HAMPs 2 to 5 (red highlights in Figure 1). The mutations leading to low resistance to iprodione without cross-resistance to pyrrolnitrin and phenylpyrroles (I365S, I365S V239, shaded in green in Figure 1) affect HAMPs 1 and 3, although the major phenotypic affect is probably due to I365S in the N-terminus of HAMP3. The third phenotypic category (moderate resistance to iprodione and osmosensitivity) represented by the mutations E529A, M427T, highlighted in pink in Figure 1, and found in association with I365S, affect helices AS2 of HAMP3 and HAMP4.

Homology-modeling analyses were performed on the SWISS-MODEL workspace [45] based on the structure 3lnrA of the poly-HAMP domains from the *Pseudomonas aeruginosa* receptor Aer2 [35]. All six HAMP domains have a structure similar to that presented in Figure 1, (see Figure S1). AS1 and AS2 can be easily distinguished and are separated by the connector encompassed between the two conserved glycine residues. The AS2s of all HAMPs have a perfect helical structure even C-terminal of the conserved fraction (gray box in Figure 1). In contrast, the helical structures N-terminal of AS1s are not perfectly conserved. The structures of HAMP2, 3 and 4 look highly similar, whereas those of HAMP1, 5 and 6 show differences especially in and N-terminal of AS1. In the case of HAMP1, no helix N-terminal of AS1 was predicted. In HAMP5 the helical part of AS1 seems shorter, and in HAMP6, the helical part of AS1 is shifted towards the N-terminus. The last four residues preceding the conserved glycine residue, namely AATD, do not seem to form a helix.

### Impact of the Bos1 modifications on HAMP structures

We then compared the predicted structures of the mutated HAMP domains to the corresponding wild-type domains. Table 3 summarizes the differences observed between the mutant and the wild-type peptides. Briefly, all modifications replacing a hydrophobic, aliphatic residue by a polar or aromatic residue impacted the AS1 and/or AS2 helical structures, whereas the replacements of polar amino acids did not produce any change in the structure predictions (e.g., T581P, E529A). It became evident that all structural changes affected either the HAMP specific helixes AS1 helix or AS2. We therefore focused our analyses on these regions supposing that the interactions of both could be important for Bos1 function in signal transduction. When zooming the molecular models on the AS1 and AS2 helices proximal to the connector, one can identify for each HAMP, pairs of four to five amino acids facing each other, respectively between AS1 and AS2 (amino acids highlighted in yellow in Figure 1 and pointed out in Figures 2 and 3). The 2D representation of these helices (Figure 4) shows an identical arrangement and a strong sequence conservation of the putatively interacting hydrophobic residues in the four HAMP domains.

**Figure 2:**
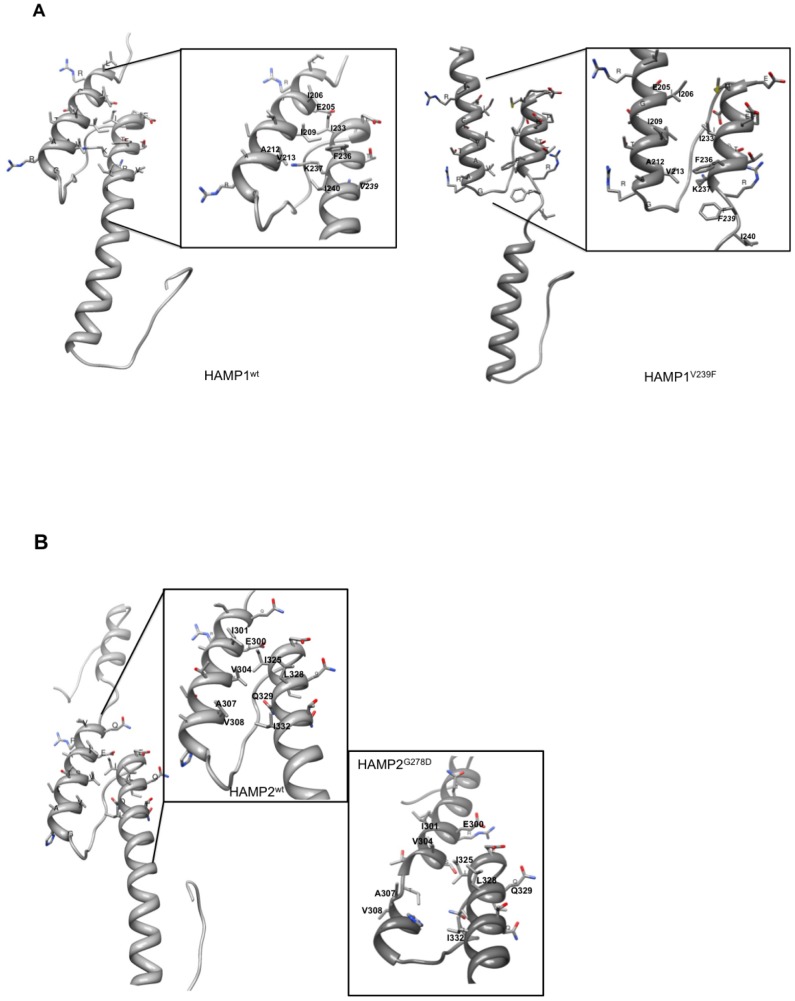
Homology based models of HAMP domains with focus on interactions between AS1 and AS2. A/ HAMP1 wild-type (wt) and mutant peptides. B/ HAMP2 wt and mutant peptides. The orientation of the peptides is from up (N-terminus) to down (C-terminus). Solely, the side chains from amino-acids of the connector proximal helices are displayed. Residues potentially involved in interactions are labelled; modified residues are written in italics.

**Figure 3:**
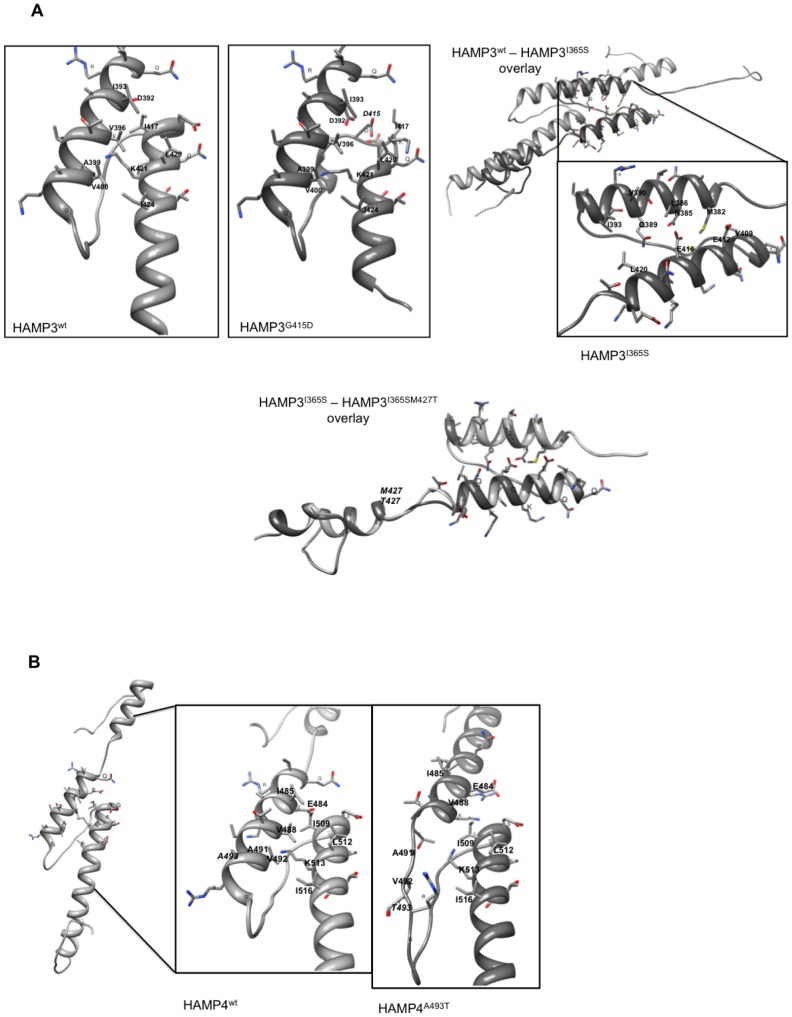
Homology based models of HAMP domains with focus on interactions between AS1 and AS2. A/ HAMP3 wt and mutant peptides. In the last panel I365S (light grey) and I365S M427T (dark grey) isoforms are shown in an overlay. B/ HAMP4 wt and mutant peptides. The orientation of the peptides is from up (N-terminus) to down (C-terminus) or from right to left (A). Solely, the side chains from amino-acids of the connector proximal helices are displayed. Residues potentially involved in interactions are labelled; modified residues are written in italics. In overlaid models, the wild-type peptide is shaded in light grey, the mutant peptide in dark grey.

**Figure 4:**
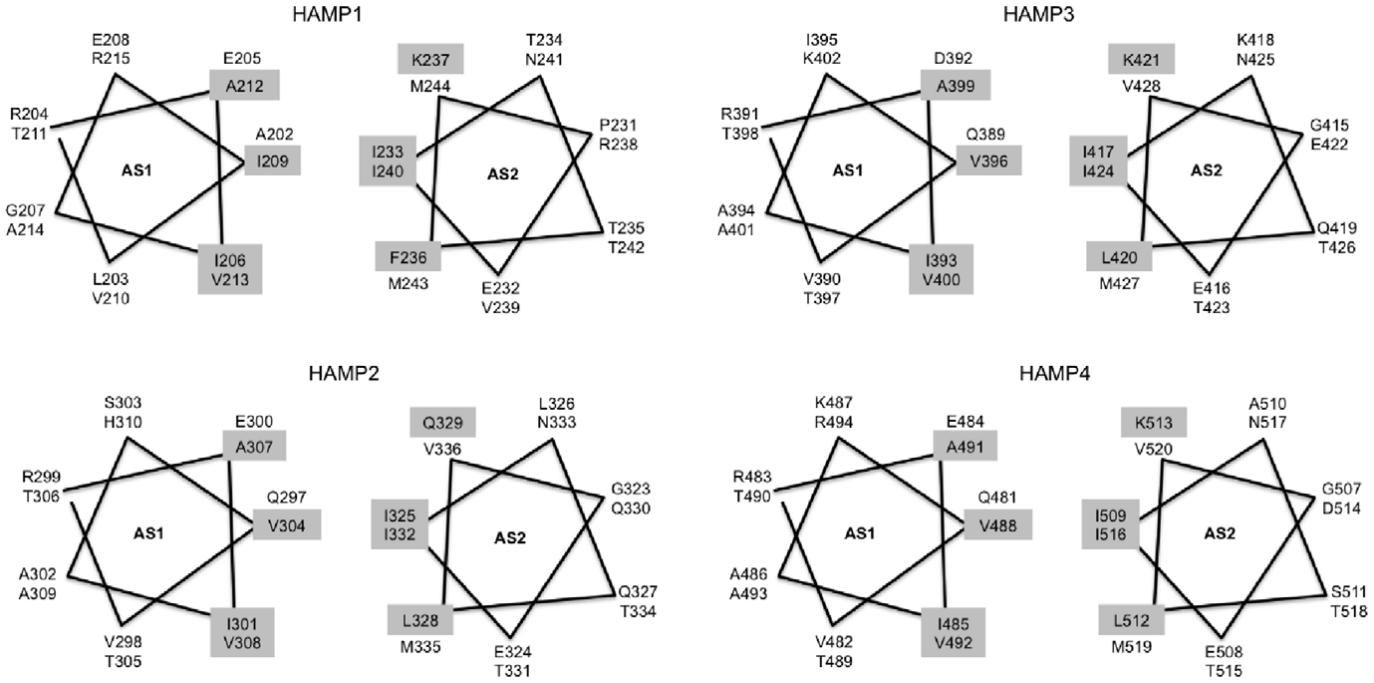
2D-schematic representation of the helical arrangement of connector proximal helices from HAMP domains 1–4. Two helical turns of seven amino acids each are listed. Both helices are facing each other such as in the model of Figure 1. The grey shading highlights interacting residues. AS1 = amphipathic helix 1; AS2 = amphipathic helix 2.

Figures 2 and 3 show the structural changes observed for the validated mutations. No structural changes were observed for HAMP5 with the selected mutants regardless of the model structure (3lnrA or 1qu7A) used (Table 3 and data not shown).

#### V239F

This mutation – observed in combination with I365S - replaces the aliphatic valine, located in the interacting region of AS2 of HAMP1, by the aromatic residue phenylalanine. The replacement abolishes the helical structure C-terminal of F239. Although the HAMP1 wt and mutant structures do not perfectly superimpose, most of the hydrophobic interactions seem to be maintained, such as those between amino acids I209 and I233, between A212, V213 and F236, K237. Only, the interacting amino acid I206 has been displaced from I233 (Figure 2A).

#### G278D

The replacement of glycine 278, located N-terminal of HAMP2 AS1, by the aspartic acid residue leads to a predicted perturbation of the AS1 helical structure distancing I301 and V308 from interaction (Figure 2B).

#### G415D

This mutation affects the conserved glycine between the connector and AS2 in HAMP3, by an aspartic acid residue instead of a cysteine. Its impact seems limited to HAMP3 AS2, perturbing the connector proximal helix and displacing I417 from interaction (Figure 3A).

#### I365S

Although the model of HAMP3 isoform I365 is not well supported (QMean Z score −4.18) by the alignment to the 3lnrA structure, one consequence of this amino acid replacement shown in Figure 3A is that both helical structures are shifted towards the N-terminus. The first helical structure is predicted between A381 and I393 instead of AS1 between L386 and K402. The second helix starts at Q407 in the middle of the connector sequence. Given the observed distance between both helices, the interacting residues are not the same as in wt HAMP3. Instead of I393, V396, A399 and V400 the interacting residues in the first helix seem to be M382, N385, L386, Q389, and I393. According to the model, they may interact with residues V409, E412, E416, and L420 of the second helix.

#### I365S M427T

The mutation of this hydrophobic residue located in AS2 by the polar threonine in HAMP3 harbouring already the I365S replacement, only produces a minor impact on AS2 compared to HAMP3^I365S^, visible in the overlay of both predicted structures (Figure 3A, lower panel).

#### A493T

The replacement of the hydrophobic alanine in HAMP4's AS1 by the polar threonine abolishes the C-terminal helical structure of AS1, displacing the interacting residues A491 and V492 (Figure 3B).

#### T581P

No visible changes were observed for HAMP5 structure after the replacement of the polar threonine by the hydrophobic proline. However, the predictions for HAMP5 and HAMP6 based on the 3lnrA crystal structure are less well supported than those of the other HAMP domains (QMEAN Z-Score: −4.42, according to [55]). Consequently, we cannot exclude an impact of the mutation T581P on HAMP5 structure *per se*.

## Discussion

Phenylpyrrole and dicarboximide fungicides affect the fungal osmotic signal transduction cascade. Although their precise mode of action remains unclear, Pillonel and Meyer [30] showed differences in protein kinase inhibition profiles between phenylpyrrole and dicarboximide fungicides. In many plant pathogenic fungi, isolates cross-resistant to both fungicides harbour mutations in the osmo-sensing histidine kinase gene (reviewed in [52]). In this study we compared the selection exerted by the dicarboximide iprodione to that exerted by pyrrolnitrin, a natural and structural analogue of phenylpyrroles, on the function and structure of the histidine-kinase Bos1 of *B. cinerea*.

Given the mutants analyzed, different phenotypic categories were selected. Firstly, two parental strains (BC1 and BC26) displayed low resistance to the dicarboximide iprodione without cross-resistance to phenypyrroles or pyrrolnitrin as well as no sensitivity to hyperosmotic conditions. This phenotype matches the previously described ImiR1 phenotype observed among field populations of *B. cinerea* [18], [19], [56]. Secondly, the mutants selected on pyrrolnitrin showed high levels of cross-resistance to dicarboximides and phenylpyrroles including pyrrolnitrin, as well as osmosensitivity. This phenotype matches the ImiR4 phenotype selected only under laboratory conditions [15], [16], [56], [57]. Moreover, it resembles the *bos1* loss-of-function phenotype observed after gene replacement [20], [22], which is unable to transmit the signals derived from phenylpyrroles, dicarboximides and hyperosmotic stress. Mutants isolated on iprodione, with high resistance levels to this dicarboximide, presented low to moderate resistance to pyrrolnitrin and phenylpyrroles, as well as osmosensitivity. Mycelial growth of all selected mutants was clearly affected, no matter the resistance profile. This equally held true for conidiation and aggressiveness on plant tissue. These findings point to a fitness cost associated with the acquisition of fungicide resistance.

The results were comparable between the iprodione-induced mutants from this study and the pyrrolnitrin-induced mutants previously reported [13]. Reduced fitness has been suggested as a possible explanation for the absence of field isolates highly resistant to dicarboximides and phenylpyrroles [2]. There is a practical consequence. If pyrrolnitrin and iprodione cross-resistance occurred in the field, it would have a detrimental effect on the fitness in terms of viability and aggressiveness of the resistant mutants. In turn, the sensitive strains, would gain ground once the fungicides are no longer active. All together, this would ensure the lifespan of phenylpyrroles or of pyrrolnitrin-producing biological control agents.

Seeking to correlate the phenotypic changes to structural modifications in the signal transduction HK Bos1, we first set out to analyze the sequence of the *bos1* gene. In most isolates, we found mutations, from nonsense mutations (G81STOP) – leading to the expected loss-of-function phenotype – to amino-acid replacements in the HAMP domains potentially involved in ST. In most cases, strains with identical mutations showed similar resistance profiles to the dicarboximide iprodione and to the phenylpyrroles suggesting that the mutations could be responsible for the phenotypes. Secondly, using site directed mutagenesis of the *bos1* gene, we were able to test eight of the twelve sequenced point mutations. By comparing these resistance profiles to those of the corresponding *in vivo* selected mutants, we confirmed the role of the following mutations: I365S leading to an ImiR1 phenotype and G278D, G415D, I365S T581P, A493T leading to loss-of-function phenotypes. The *in vitro* mutants with the genotypes I365S V239F, I365S M427T, I365S E529K displaying low to moderate resistance to iprodione, are not in agreement with the phenotypes observed for the *in vivo* mutants (BC1G20I3, H6G20I2/I3, BC26G20I1/I3), which are highly resistant to iprodione associated with moderate resistance to phenylpyrroles. We suspect that additional mutations responsible for higher resistance levels within these genetic backgrounds were selected on high iprodione concentrations.

We confirmed that the A493T mutation confers moderate levels of resistance to phenylpyrroles and iprodione, but as the strains BC25G20I2 and BC25G20I3 harboring this mutation show different resistance levels to phenylpyrroles. We can therefore hypothesize that at least strain BC25G20I3 carries an additional mutation leading to higher resistance levels to these compounds.

To sum up the molecular data of our mutational analysis: i/ all HAMP domains were affected by modifications inducing resistance. Only the mutation of V239F in HAMP1 did not modify the resistance profile in addition to the I365S mutation present in the initial strain; ii/ the replacement of conserved glycine residues (G278D, G415D) by charged amino acids led to ImiR4 (loss-of-function) phenotypes; iii/ mutations with a low impact on the resistance levels (highlighted in green or purple in Figure 1) localized outside AS1, whereas mutations leading to ImiR4 phenotypes affected either AS1 or AS2. Of particular interest are the mutations E529A and M427T (highlighted in purple in Figure 1), which led to osmosensitivity in the I365S background. They only weakly interfered with sensitivity to phenylpyrroles, but these replacements seem to abolish osmotic ST.

Concerning the structural changes predicted for the HAMP domains and the related phenotypes, our study provides first insights:

Generally, only the modifications of hydrophobic residues impact the helical structures of the HAMP domains, whereas the replacements of polar residues do not seem to interfere with them. We suspect that these perturbations of the helical structures abolish or modify the supposed interactions between AS1 and AS2. The strongest phenotypes observed (loss of function) correlate with the loss of two interacting residues in HAMP2 (mutant G278D), one in HAMP3 (mutant G415D) or two in HAMP4 (mutant A493T). The mutants displaying an ImiR1 phenotype (mutation I365S alone or associated with V239F) have an HAMP3 domain with modified interactions between AS1 and AS2 compared to the wt. The fact that the I365S mutant is not affected for phenylpyrroles and osmotic ST suggest that these newly created interactions in HAMP3 are sufficient to transduce these signals. Concerning the modifications that did not alter the helical structure, but lead to a modification in signal transduction (T581P, M427T, E529A), we suspect that the affected residues could be important for ST or that the replacement residues might hinder ST – at least in combination with I365S. This holds true particularly for T581P leading to a loss-of-function phenotype in association with I365S, whereas E529A and M427T could be involved principally in osmotic signal transduction.

In summary, our results suggest that mutations resulting in Bos1-loss-of-function phenotypes (those highlighted in red in Figure 1) i.e. completely disrupting signal perception and/or transduction, either abolish important interactions between AS1 and AS2 of HAMPs 2–4 or affect potential key residues in Bos1 ST, such as T581.

In order to better understand differential ST through Bos1 we were particularly interested in mutations conferring resistance to only one chemical family, dicarboximides or phenylpyrroles, or modifying osmosensitivity, because they may have a partially functional Bos1 protein. Using site-directed mutagenesis, we obtained two categories of partially functional Bos1, one leading only to low resistance to dicarboximides (I365S, ImiR1) and the other affecting also osmosensitivity (I356S E529A, I365S M427T). These modifications do not seem to abolish interactions between AS1 and AS2, potentially essential to Bos1 ST.

Altogether our data reveal Bos1 modifications that lead to loss-of-signal-transduction, principally in HAMP domains 2–5. Only changes outside AS1 and the connector domains maintained partial Bos1 function. Some modifications interfere only with dicarboximide (I365S) or osmotic ST (E529A, M427T).

Our study gives a first glimpse on structure-function relationship for differential ST through an eukaryotic HAMP-containing histidine kinase. We analyzed the HAMP structures individually, although the structure of the model protein used for our analyses involves protein dimers [35], we cannot exclude that some mutations affect intra- or inter-molecule interactions different from those we analyzed. It would be interesting to resolve the crystal structure of eukaryotic HAMP containing proteins – especially of histidine kinases with successive HAMP domains – in order to better understand the signal transduction processes regulated by these proteins.

## Supporting Information

Figure S1 **Predicted models for the HAMP domains of the histidine-kinase Bos1.** (A) front, (B) back. Model predictions were performed on the Swiss-model server [45] by alignment to the crystal structure 3lnrA of the aerotaxis receptor Aer2 of *Pseudomonas aeroginosa* [35]. The orientation of the peptides is from up (N-terminus) to down (C-terminus). The side chains of amino acids located in helical regions (A) and in the connector (B) facing the neighbouring helices are presented.
(PPTX)

Table S1 ***In vitro*** **response of pyrrolnitrin- and iprodione-induced mutants to pyrrolnitrin, iprodione, and phenylpyrroles.**
(DOCX)

Table S2 **Primers used in this study for amplification and sequencing of the** ***bos1*** **gene.**
(DOCX)
