# Supplementary figures and images for: Functional and Structural Comparison of Pyrrolnitrin- and Iprodione-Induced Modifications in the Class III Histidine-Kinase Bos1 of Botrytis cinerea
Source: PLoS One. 2012 Aug 13;7(8):e42520. doi: 10.1371/journal.pone.0042520 (PMC3418262; doi:10.1371/journal.pone.0042520)

## Slide 1
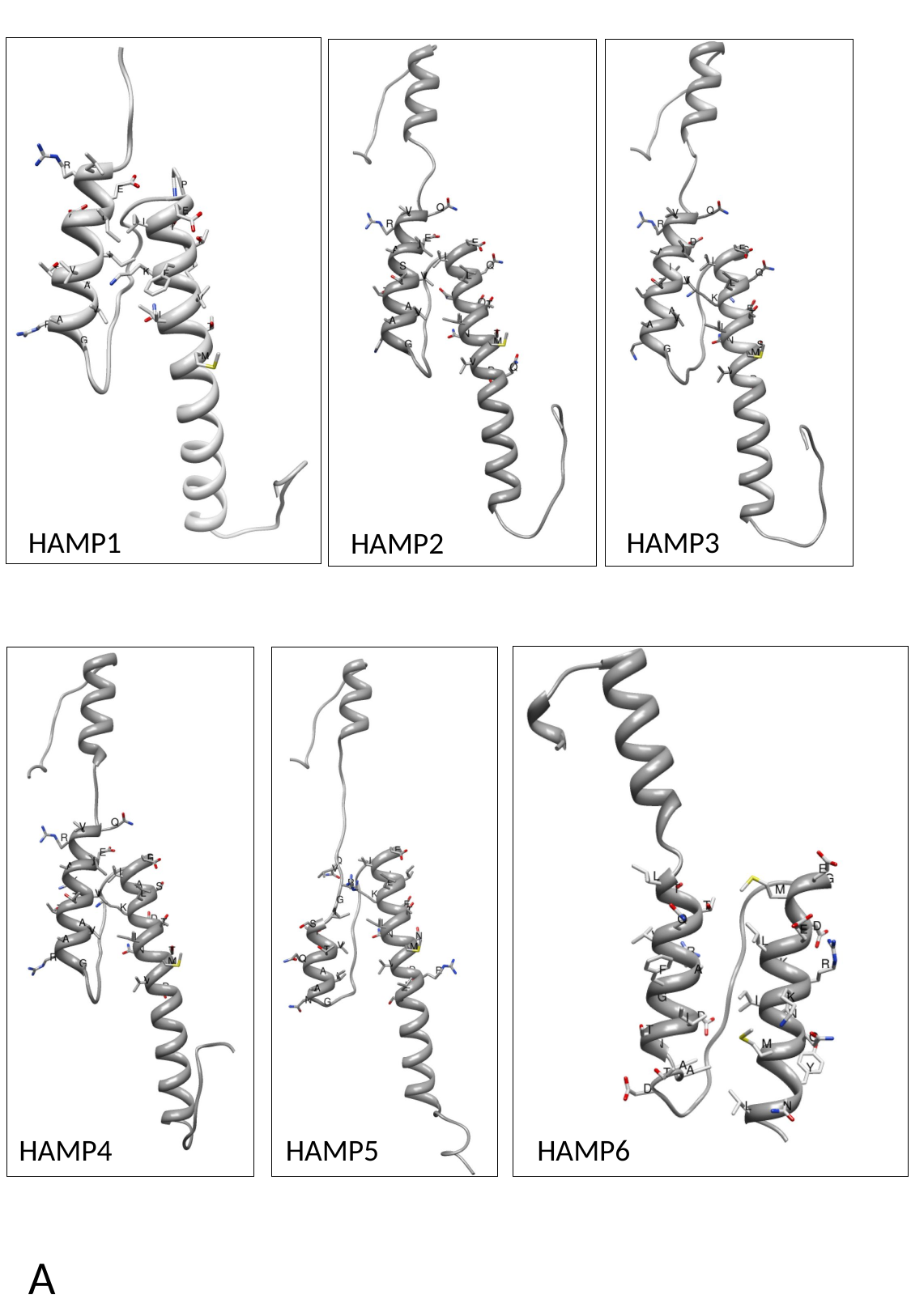

HAMP1
HAMP3
HAMP2
HAMP4
HAMP5
HAMP6
A

## Slide 2
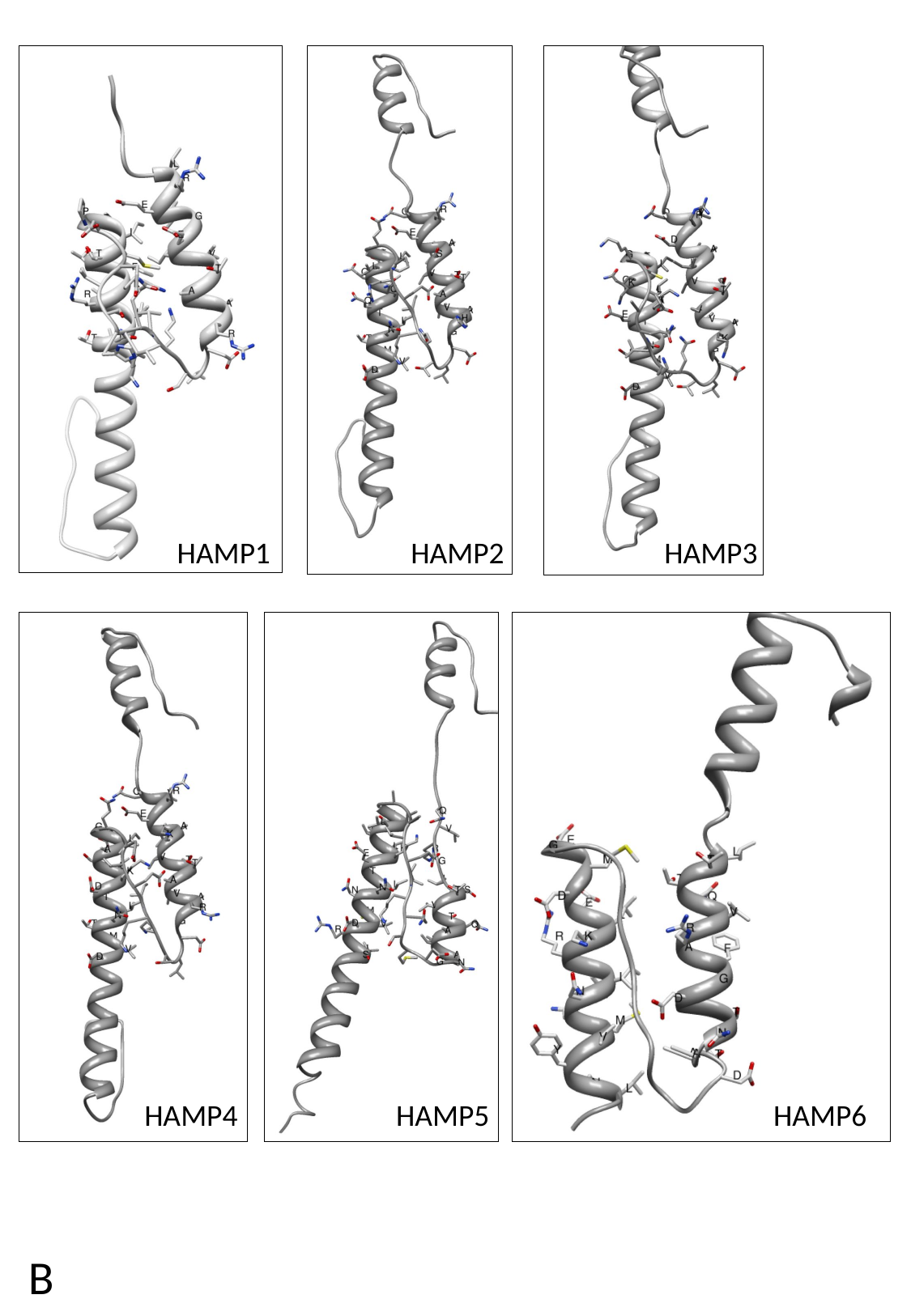

HAMP1
HAMP2
HAMP3
HAMP4
HAMP5
HAMP6
B

Supplement: Figure S1 — Predicted models for the HAMP domains of the histidine-kinase Bos1. (A) front, (B) back. Model predictions were performed on the Swiss-model server [45] by alignment to the crystal structure 3lnrA of the aerotaxis receptor Aer2 of Pseudomonas aeroginosa [35]. The orientation of the peptides is from up (N-terminus) to down (C-terminus). The side chains of amino acids located in helical regions (A) and in the connector (B) facing the neighbouring helices are presented. (PPTX) [file pone.0042520.s001.pptx]
