# Supplementary material for: Functional and Structural Comparison of Pyrrolnitrin- and Iprodione-Induced Modifications in the Class III Histidine-Kinase Bos1 of Botrytis cinerea
Source: PLoS One. 2012 Aug 13;7(8):e42520. doi: 10.1371/journal.pone.0042520 (PMC3418262; doi:10.1371/journal.pone.0042520)
Supplement: Table S1 — In vitro response of pyrrolnitrin- and iprodione-induced mutants to pyrrolnitrin, iprodione, and phenylpyrroles. (DOCX) [file pone.0042520.s002.docx]

**Table S1.**  *In vitro* response of pyrrolnitrin- and iprodione-induced mutants to pyrrolnitrin, iprodione, and phenylpyrroles.

| Isolate | Transfer generation^a^ | Pyrrolnitrin | Iprodione | Fenpiclonil | | Fludioxonil |
| --- | --- | --- | --- | --- | --- | --- |
|  |  |  | (Dicarboximides)^b^ | (Phenylpyrroles) | | |
| BC1 | G0 | 0.005 | 6 | 0.1 | 0.03 | |
|  | G20C | 0.005 | 6 | 0.1 | 0.03 | |
|  | G20P^c^ | 8.7 | >25 | >10 | >10 | |
|  | G20I1 | 0.2 | >25 | 3 | 1.5 | |
|  | G20I2 | >0.5 | >25 | 3 | 1.5 | |
|  | G20I3 | 0.03 | >25 | 3 | 1.5 | |
|  |  |  |  |  |  | |
| BC21 | G0 | 0.007 | <2.5 | 0.05 | 0.02 | |
|  | G20C | 0.007 | <2.5 | 0.05 | 0.02 | |
|  | G20I1 | >0.5 | >25 | 5.5 | 10 | |
|  | G20I2 | >0.5 | >25 | 0.3 | 0.1 | |
|  | G20I3 | >0.5 | >25 | nd^d^ | nd | |
|  |  |  |  |  |  | |
| BC25 | G0 | 0.005 | 1 | 0.06 | 0.02 | |
|  | G20C | 0.005 | 1 | 0.06 | 0.02 | |
|  | G20P | 9.0 | >25 | 9 | 10 | |
|  | G20I1 | >0.5 | >25 | 2.2 | 10 | |
|  | G20I2 | 0.4 | >25 | 1 | 0.8 | |
|  | G20I3 | 0.4 | >25 | 3.8 | >10 | |
|  |  |  |  |  |  | |
| BC26 | G0 | 0.006 | 10 | 0.03 | 0.03 | |
|  | G20C | 0.006 | 8 | 0.03 | 0.03 | |
|  | G20P | 7.4 | >25 | >10 | >10 | |
|  | G20I1 | 0.08 | >25 | 1 | 0.9 | |
|  | G20I2 | >0.5 | >25 | nd | nd | |
|  | G20I3 | 0.1 | >25 | 1 | 0.3 | |
|  |  |  |  |  |  | |
| H6 | G0 | 0.001 | 1.5 | 0.02 | 0.02 | |
|  | G20C | 0.001 | 1.5 | 0.02 | 0.02 | |
|  | G20P | 4.6 | >25 | 10 | 10 | |
|  | G20I2 | 0.06 | >25 | 0.3 | <0.1 | |
|  | G20I3 | 0.06 | >25 | 0.4 | 0.2 | |

^a^ G0 is the wild-type parent isolate, G20C is the 20^th^ transfer generation produced on PDA medium (control), G20P is the the 20^th^ transfer generation produced on PDA amended with pyrrolnitrin and G20I is the 20^th^ transfer generation produced on PDA amended with iprodione.

^b^ The EC_50_ values were determined on mycelial growth for pyrrolnitrin and on germ tube elongation for dicarboximides and phenylpyrroles as explained in the Materials & Methods section..

^c^ for G20P, indicated values are means of the values obtained for the three pyrrolnitrin-induced mutants according to Ajouz et al. (2010b)

^d^ nd = not determined
