# Supplementary material for: Functional and Structural Comparison of Pyrrolnitrin- and Iprodione-Induced Modifications in the Class III Histidine-Kinase Bos1 of Botrytis cinerea
Source: PLoS One. 2012 Aug 13;7(8):e42520. doi: 10.1371/journal.pone.0042520 (PMC3418262; doi:10.1371/journal.pone.0042520)
Supplement: Table S2 — Primers used in this study for amplification and sequencing of the bos1 gene. (DOCX) [file pone.0042520.s003.docx]

**Table S2**: Primers used in this study for amplification and sequencing of the *bos1* gene.

| **primer-name** | **position** |  | **sequence** |
| --- | --- | --- | --- |
| bcos1prom1 | 16..35 | F | CCCCTGTGATTAAACCGAGT |
| daf3'RP | 102..126 | R | TAGCTTTTTGGGATTGGGACTTTGG |
| bos1_promLP1 | 229..250 | F | CTAGCTGCCGACCGACACAGGT |
| bos1-F1 | 749..769 | F | GGCATTATCGCTTCGTAGTGG |
| bos1_promLP2 | 959..980 | F | CTCACTCATTCACGCACGCACA |
| bos1_promRP1 | 1079..1100 | R | GTTACCCGCTTCGACGGTGTTG |
| bos1-F2 | 1203..1223 | F | CTACTGCGATCCTGCAAACTC |
| daf5'1 | 1277..1303 | R | TGCCGTATCTGCACCTGGTAACCTAAT |
| bos1-F3 | 1721..1742 | F | GTAGCAGCTCTCGAAAGGGAAC |
| bos1-F4 | 2070..2091 | F | GGAAGGAGTTGACTGACAATGG |
| Ham3 | 2021..2044 | R | GGCTTGTCCACCGAGAATACCTTC |
| I365_LP | 2248..2269 | F | TGAGAGACCAGCCCAGGGTGAG |
| bos1-R4 | 2608..2629 | R | CTCCCTAGCAATCTTCGTGACT |
| I365_RP | 2753..2774 | R | CGACAGCGGTGGTAACCTTTGC |
| bot1' | 2942..2971 | R | GTCTTTCCACTTTCCTTCGACGTTATCAAC |
| bos1-R3 | 3070..3090 | R | GCAGCAGCAACCTCAATCTTC |
| His1 | 3073..3095 | F | GATTGAGGTTGCTGCTGCGGGTG |
| bos1-R2 | 3515..3536 | R | GAGTGGTACTGACAGGTGTTCG |
| bos1-F5 | 3575..3597 | F | CATTACGCCGAGTGCCTTCTCAA |
| bos1-R1 | 3993..4013 | R | ACCACGTGATCTGGAACTGAG |
| His3 | 4369..4387 | F | CGCCTCGCAACCTCAGATA |
| Hav2 | 5044..5071 | F | GTAGGGAGGATTCGAAGCAACCGCTAAG |
| H4 | 5231..5251 | R | ACCTCCAAGGGTTGCACATTT |
| bos1-R5 | 5570..5592 | R | ATCGAACTTCGGCCACGATGAAT |
| BcOS1term1 | 5706..5725 | R | TCGCCATTCTGAAAACAGGT |

F=forward; R=reverse
